# The relationships between microbiome diversity and epidemiology in domestic species of malaria-mediated mosquitoes of Korea

**DOI:** 10.1038/s41598-023-35641-3

**Published:** 2023-06-05

**Authors:** Jeong Hyeon Lee, Hyun-Woo Kim, Bilal Mustafa, Hee Il Lee, Hyung Wook Kwon

**Affiliations:** 1 Department of Life Sciences, Incheon National University, 119 Academy-ro, Yeonsu-gu, Incheon, 22012 Republic of Korea; 2 Convergence Research Center for Insect Vectors, 119 Academy-ro, Yeonsu-gu, Incheon, 22012 Republic of Korea; 3 Division of Vectors and Parasitic Diseases, Korea Disease Control and Prevention Agency, 187 Osongsaenmyeong2-ro, Osong-eup, Heungdeok-gu, Chunbuk, Cheongju, 28159 Republic of Korea

**Keywords:** Computational biology and bioinformatics, Ecology, Microbiology

## Abstract

Microbiota in the mosquito plays an important role in their behavior and vector competence. The composition of their microbiome is strongly influenced by the environment, especially their habitat. The microbiome profiles of adult female *Anopheles sinensis* mosquitoes from malaria hyperendemic and hypoendemic areas in Republic of Korea were compared using 16S rRNA Illumina sequencing. In different epidemiology groups, the alpha and beta diversity analyses were significant. The major bacterial phylum was Proteobacteria. The most abundant species in the microbiome of hyperendemic mosquitoes were the genera *Staphylococcus*, *Erwinia*, *Serratia*, and *Pantoea*. Notably, a distinct microbiome profile characterized by the dominance of *Pseudomonas synxantha* was identified in the hypoendemic area, suggesting a potential correlation between the microbiome profiles and the incidence of malaria cases.

## Introduction

Malaria, which is caused by the protozoan parasite *Plasmodium* which infects mosquitoes and human, spreads the infection to its mammalian hosts by the bite of *Plasmodium*-infected anopheline mosquitoes^1^. Generally, vaccines such as Mosquirix against malaria are currently offered to the public^2^. However, the economic costs of the vaccine must still be considered. Subsequently, most intervention efforts focus on controlling mosquito populations, typically using chemical pesticides. Abusive use of several chemical pesticides resulted in resistance in mosquito populations^3^ requiring the use of alternative mosquito control strategies including manipulation of mosquito-microbiome^4–6^.

The microbiome is an ecosystem of commensal, symbiotic, and pathogenic bacteria that interact with a host^7^. Mosquitoes as natural hosts also contain a diverse range of microorganisms including bacteria, fungi, and viruses^8^. Among these, bacteria are continuously interacting with mosquitoes^9–11^ and influence the nutrition, development, immunity, and behaviors of host mosquitoes. For instance, infection with the bacterial endosymbiont *Wolbachia pipientis* prevents numerous arbovirus infections^12,13^. Indeed, the introduction of *wMel* strain of *Wolbachia pipientis* into the *Aedes aegypti* population turned out to be effective in reducing the incidence of symptomatic dengue and the case of hospitalizations by dengue fever^14^. Moreover, *Chromobacterium* sp. exposure causes high mortality in larval and adult mosquitoes and reduces mosquitoes’ susceptibility to malaria and dengue infection^15^, suggesting that specific microbiota in mosquitoes can alter susceptibility to disease infection^16^.

Along with physiological interactions, the bacterial composition of mosquitoes collected from natural habitats is highly variable depending on the geographical origin and ecology^17–20^. Recently, *Anopheles* mosquitoes collected in the field reveal greater levels of inter-mosquito heterogeneity in community composition^21^. However, a thorough investigation of the relationships between regional malaria incidence and microbiome profiles is still absent. This study presents a comparative analysis of the microbiome profiles in *Anopheles sinensis* mosquitoes collected from malaria-endemic and malaria-free areas in the Republic of Korea (ROK). *Plasmodium vivax* malaria in ROK was officially eradicated by World Health Organization (WHO) in 1979^22^. However, malaria re-emerged in 1993 in a region on the border with North Korea where 300–500 malaria cases have been reported every year^23^. It is still elusive whether the reemergence of malaria cases result from specific natural habitats and long-range migration from malaria hyperendemic area from northern regions. Malaria incidence in border regions is higher than in other areas due to factors such as restricted access to healthcare, the propensity of marginalized groups-who generally live in border regions-to seek treatment, and difficulties distributing prevention initiatives in hard-to-reach populations^24^. It will be difficult to eradicate malaria in border regions, but improved surveillance is the key to finding the source of any novel imports or re-introductions. Thus, the microbiome profile patterns of mosquitoes in various regions need to be compared and analyzed to monitor malaria outbreaks. Therefore, the focus of this study is on the identification of microbial diversity in female *An. sinensis* mosquitoes from malaria-free or hypoendemic areas and hyperendemic areas where sporadic malaria cases were reported. By using metagenomics analysis, this study delves into determining whether *An. sinensis* in malaria-endemic areas possess distinct microbiome profiles.

## Results

### Microbiome profiles of *Anopheles sinensis* female in Korea

**Figure 1:**
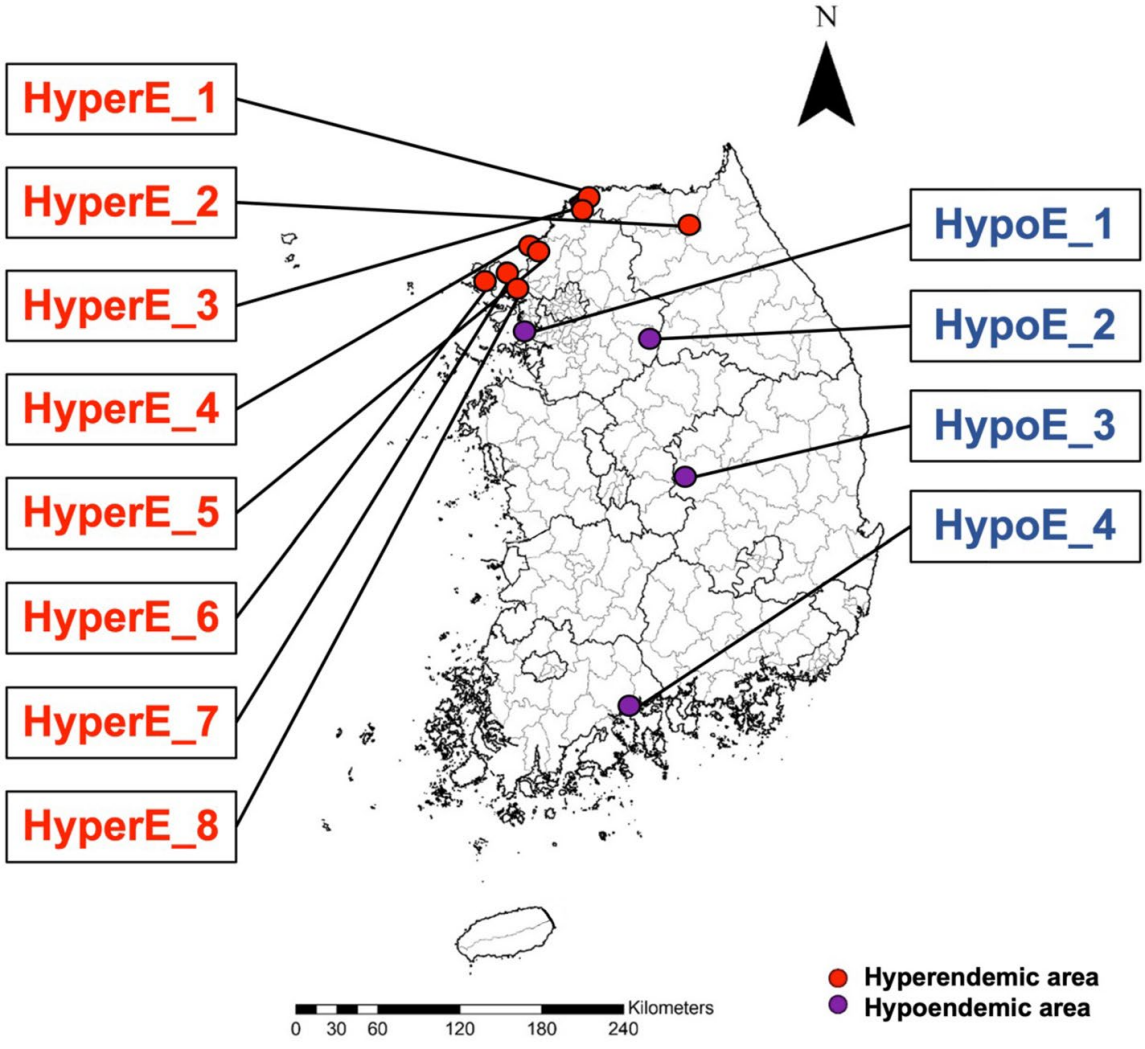
Mosquito collecting site The mosquito collecting sites were selected based on the incidence of malaria cases. The number of malaria patients per 100,000 was used to divide hyperendemic (HyperE) and hypoendemic (HypoE) areas. The collecting site were determined using statistics of malaria patients. The analysis could have improperly accounted for habitat or ecology since each region was not ecologically analyzed. Hypoendemic areas 1 and 2 were selected as areas near the hyperendemic area among the places where malaria did not occur to assess the impact of epidemiology rather than geographical features of the microbiome profile of mosquitoes (HyperE-1: 348-19, Daemari Myojang-ro, Cheorwon-eup, Cheorwon-gun, Gangwon-do HyperE-2: 439-3 Guam-ri, Nam-myeon, Yanggu-gun, Gangwon-do HyperE-3: 31, Dodaero 12beon-gil, Daegwang-ri, Sinseo-myeon, Yeoncheon-gun, Gyeonggi-do HyperE-4: JSA Daeseong-dong Civil Service Class, Josan-ri, Gunnae-myeon, Paju-si, Gyeonggi-do HyperE-5: 119, Tongilchon-gil, Baekyeon-ri, Gunnae-myeon, Paju-si, Gyeonggi-do HyperE-6: 10, Samsanbuk-ro 437beon-gil, Seokmori, Samsan-myeon, Ganghwa-gun, Incheon HyperE-7: 134 Yeonmijeong-gil, Wolgot-ri, Ganghwa-eup, Ganghwa-gun, Incheon HyperE-8: 83-23, Yulsaeng-ro, Daegot-myeon, Gimpo-si, Gyeonggi-do HypoE-1: 564 Cheongna-dong, Seo-gu, Incheon HypoE-2: 283-2, Sinjeop-ri, Buknae-myeon, Yeoju-si, Gyeonggi-do HypoE-3: Mountain 110, Seonyo-ri, Sangju-si, Gyeongsangbuk-do HypoE-4: 36, Hakdong-gil, Suncheon-si, Jeollanam-do).

A total of 60 adult female *An. sinensis* mosquitoes were collected from 12 different areas across Korea (5 mosquito samples per area) (Fig. 1). Mid-gut and salivary glands were dissected from each mosquito, then DNA was extracted. A total of 1,936,902 sequences were generated from 24 samples. The average number of raw sequence reads was 80,704 (between 29,566 and 97,799 reads).

**Figure 2:**
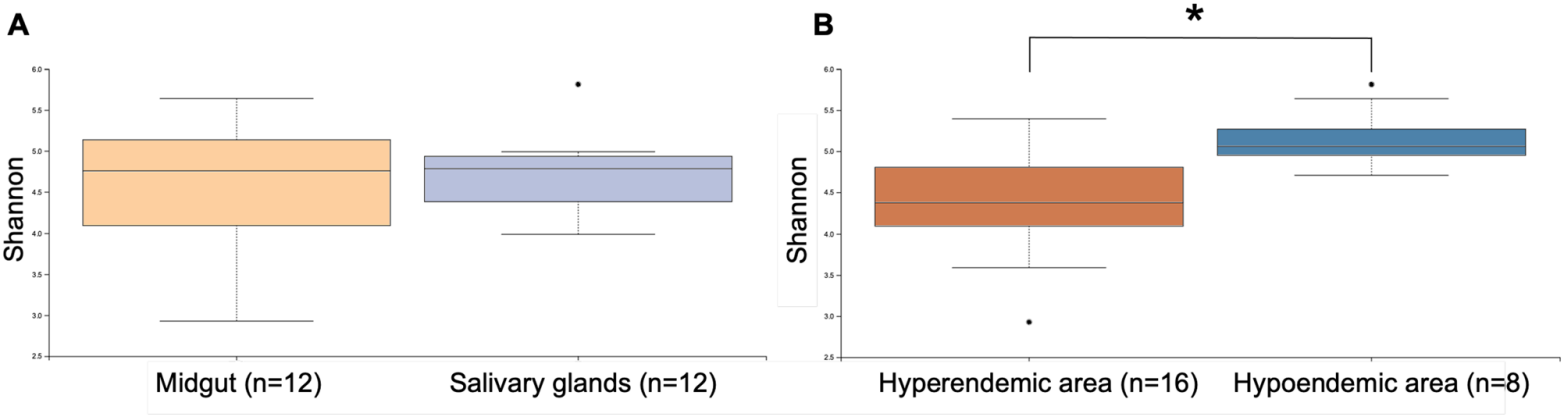
Pairwise alpha diversity comparisons of the microbiome of *An. sinensis* from different types of (**A**) organ and (**B**) epidemiology. A comparison of Shannon diversity in hyperendemic and hypoendemic areas revealed significant differences (H = 10.53, p-value = 0.001, q-value = 0.001). Kruskal–Wallis tests with Benjamini–Hochberg FDR correction were used to make these comparisons (q-value). The significance level was set at $\hbox {q}<0.05$, with n = the number of samples processed and each pool containing 5 individual mosquitoes.

Metrics of alpha diversity were subsequently assessed. (Supplementary Information 1). Alpha diversity was used to analyze the microbial communities. The Shannon index was used to measure alpha diversity. The highest Shannon diversity index was hypoendemic 3 mid-gut (M) (3.27), followed by hyperendemic 6M (2.32) and hypoendemic 2 salivary glands (S) (2.29). The alpha diversity of all Shannon diversity-evaluated samples was analyzed by organ and epidemiology (Fig. 2). There were significant differences between hyperendemic and hypoendemic areas.

**Figure 3:**
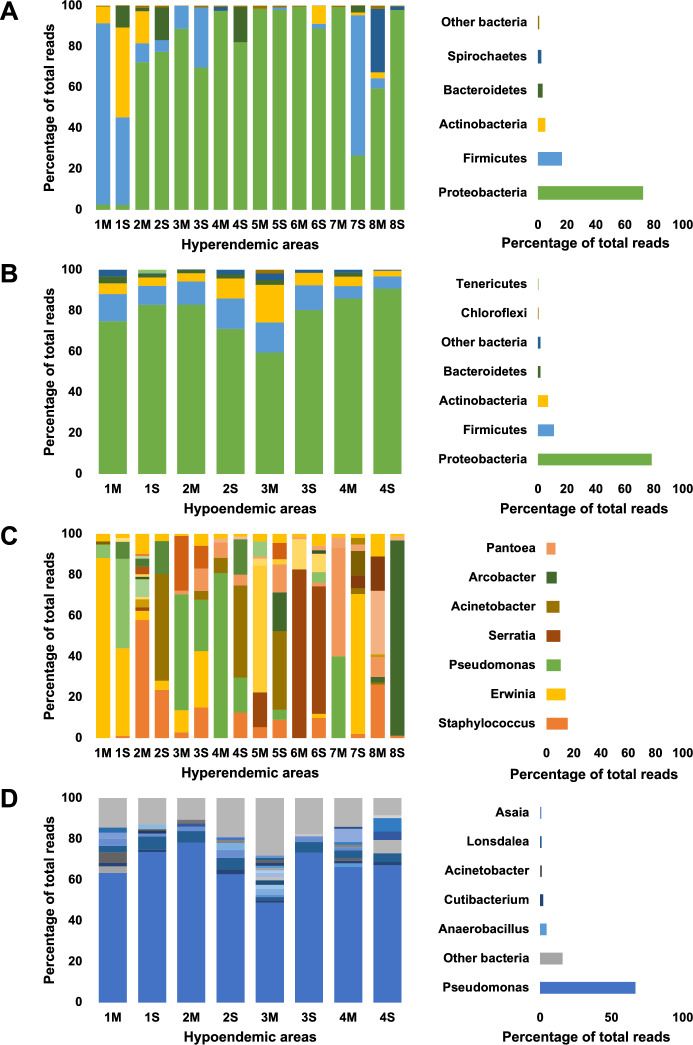
Microbiome profiles of adult *Anopheles sinensis* mosquitoes at phylum and genus levels. Left side-bar plots represent the microbiome profiles of adult female *Anopheles sinensis* mosquitoes collected from 12 regions which are divided into hyperendemic (**A**: phylum level, **C**: genus level) and hypoendemic (**B**: phylum level, **D**: genus level) areas. Each bar represents the microbiome profile of pooled sample by organ and region (M: mid-gut, S: salivary glands). Right side-bar plots represent the average of all regions.

The microbiome profiles of mosquitoes were presented at the phylum and genus level for each region and organ (Fig. 3). The most dominant one was Proteobacteria (74.52%), followed by Firmicutes (14.67%), Actinobacteria (5.65%) and Bacteroidetes (2.57%) (Fig. 3A,B, Supplementary Information 2). These four phyla accounted for 96-99% of the total OTU in the majority of the samples (Supplementary Information 2). However, Spirochaetes (31.1%) were found in hyperendemic 8M with a high ratio (Supplementary Information 2). Firmicutes were found in hyperendemic area 1M, 1S, hyperendemic area 3M, 3S, and hyperendemic area 7S (Fig. 3A, Supplementary Information 2) with high ratio than other regions. Bacteroidetes were observed to more prevalent in hyperendemic 1S, 2S, and 4S than in other regions (Fig. 3A, Supplementary Information 2).

Data at the genus level were analyzed to identify bacterial operational taxonomic units (OTUs) present in all mosquito samples. (Fig. 3C,D, Supplementary Information 3). A total of 51 genera were found, the five most abundant bacterial genera (average abundance) were *Pseudomonas* (29.22%), *Staphylococcus* (10.5%), *Erwinia* (9.37%), *Serratia* (6.91%) and *Acinetobacter* (6.7%) (Fig. 3C,D, Supplementary Information 3). Variations in microbiome profiles were observed across different regions within the hyperendemic area. The hyperendemic area 1 nearest to the northern border had a greater *Staphylococcus* ratio than the other regions (Figs. 1, 3, Supplementary Information 3). *Brevibacterium* was found in high abundance in hyperendemic area 1S, however, this microbiome was not found in any other regions except in hyperendemic area 6S. *Staphylococcus* was also found at a higher rate than in other regions in hyperendemic area 3, which is the closest region to hyperendemic area 1. In hyperendemic area 3, *Erwinia* and *Enterobacteriaceae_uc* dominate the microbiome profile. *Erwinia* was also observed in a high ratio in the hyperendemic areas 4M and 7M. Despite being close-range regions, hyperendemic areas 4 and 5 showed distinct microbiome profiles, and there were also substantial differences between organs. *Acinetobacter* dominated the microbiome in hyperendemic area 4S, followed by *Chryseobacterium*, *Erwinia*, and *Pseudomonas*. The microbiome profile of the hyperendemic area 5M was dominated by *Enterobacteriaceae_uc*, followed by *Serratia*. *Acinetobacter* dominated the microbiome profile in hyperendemic area 5S, followed by *Arcobacter* and *Pantoea*. In hyperendemic area 6, the mid-gut and salivary glands had a microbiome profile dominated by *Serratia* and *Gibbsiella*. The microbiome profile of hyperendemic area 7 varied by organ. *Pantoea* and *Erwinia* were dominant in Hyperendemic area 7M, whereas *Staphylococcus* and *Asaia* were dominant in Hyperendemic area 7S. Hyperendemic area 8S showed a distinct microbiome profile dominated by *Arcobacter*. Hyperendemic areas 8M and 2M had similar microbiome to hypoendemic areas. *Pseudomonas* dominated microbiome profile from all hypoendemic areas (Supplementary Information 3).

At the species level, *Pseudomonas* *synxantha* (24.54%) was dominant followed by *Serratia* *ficaria* (6.52%), *Acinetobacter* *soli* (6.12%), *Arcobacter* *butzleri* (4.89%), *Staphylococcus* *aureus* (4.46%), *Pantoea* *agglomerans* (3.7%) and *Erwinia* *persicina* (3.4%) (Supplementary Information 4). In the hyperendemic area, a distinct microbiome profile varied by each region in the hyperendemic area, similar to the genus level (Supplementary Information 4). The investigation focused on identifying species biomarkers, which are species exclusively found in specific regions at the species level (Table 1). The hyperendemic areas 1, 3, 4, 6, 7, and 8 showed significant values. The region-specific microbiome was dominating in the microbiome profile of each region, such as *Serratia* *ficaria*, *Staphylococcus* *sciuri*, *Erwinia* *persicina*, *Staphylococcus* *aureus*, *Arcobacter* *butzleri*, and *Erwinia* *iniecta*.

**Table 1:** Species biomarker.

Sample	Taxon name	p-value	p-value (FDR)	Taxonomic relative abundance (%)
Hyperendemic area 1	*Staphylococcus aureus*	0.0342	0.50815	50.0184
	*Brevibacterium avium*	0.01262	0.3396	23.2605
	*Staphylococcus equorum*	0.03682	0.53963	14.7103
Hyperendemic area 3	*Erwinia persicina group*	0.02578	0.76076	40.8605
	*Enterobacteriaceae*	0.03359	0.79616	18.9266
Hyperendemic area 4	*Erwinia iniecta*	0.02578	0.58038	33.8485
	*Erwinia_uc*	0.02119	0.49879	15.3244
Hyperendemic area 6	*Serratia ficaria*	0.03359	0.42652	68.4779
	*Gibbsiella quercinecans*	0.02578	0.33312	11.9534
Hyperendemic area 7	*Staphylococcus sciuri*	0.04194	0.79094	19.5395
Hyperendemic area 8	*Arcobacter butzleri*	0.03074	0.44759	49.2067
	*AF166259_s*	0.01727	0.33253	16.3773

The Kruskal Wallis H test was used to determine species biomarkers by region ($\textit{p}<0.05$). The average microbiome profile of all samples (n = 24) was compared to the microbiome profile of each region (n = 2). Significant species biomarkers were determined in hyperendemic areas (1, 3, 4, 6, 7, 8).

### Organ-specific differences in mosquito microbiome composition

**Figure 4:**
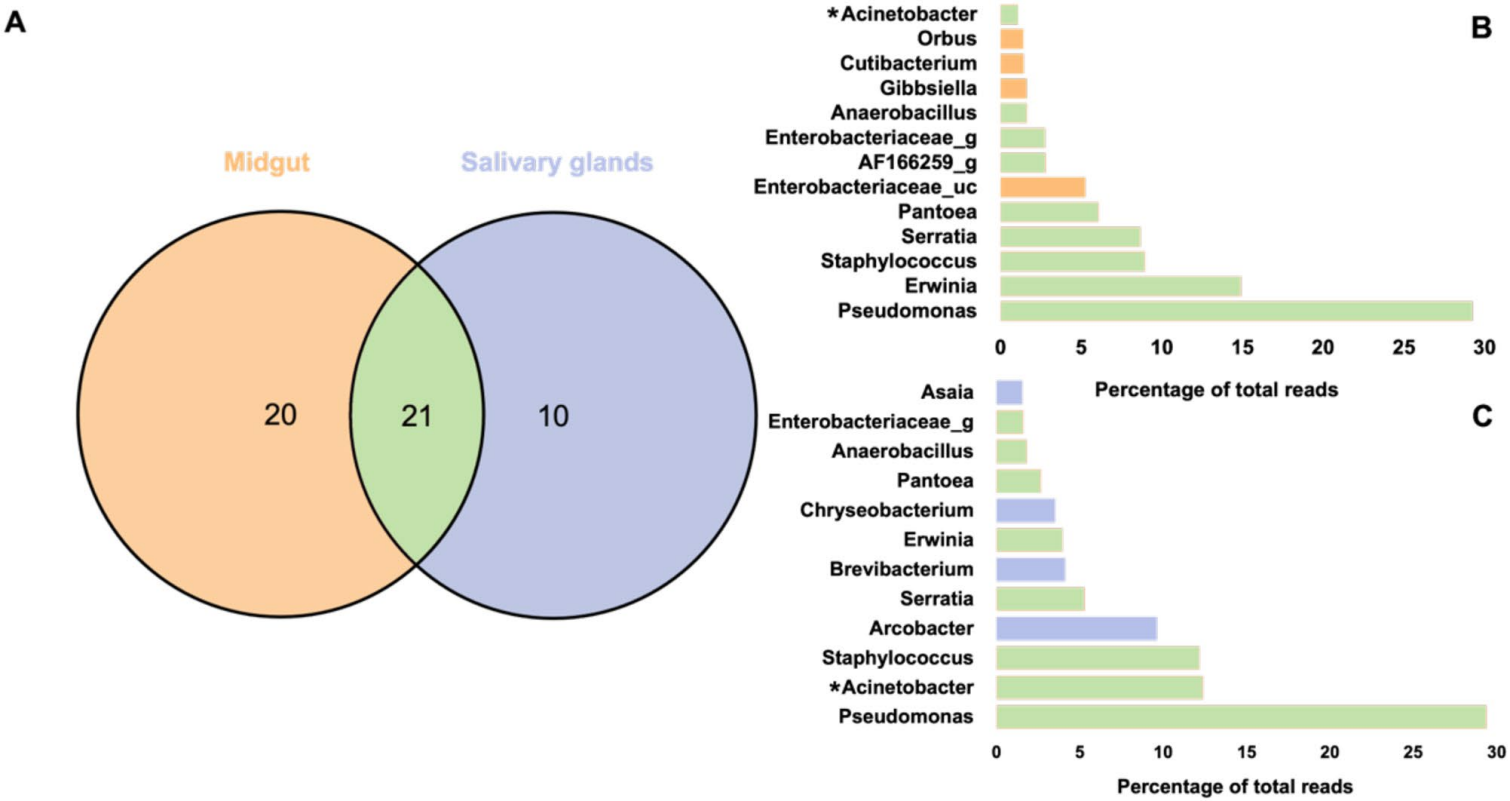
(**A**) Venn diagram analysis of shared genera by organ (**B**) Microbiome profile of mid-gut (**C**) salivary glands. There are 21 microbiotas shared by the mid-gut and salivary glands, as well as 20 and 10 organ-specific microbiotas, respectively. The microbiome shared by both organs had a high ratio, and *Arcobacter* was the only organ-specific microbiome in the salivary glands with a percentage of above 5%. In *Acinetobacter*, there was a statistically significant difference (Wilcoxon rank-sum test, $\textit{p}<0.05$).

First, The average values of the mid-gut and salivary gland microbiomes were compared across all regions (Supplementary Information 3). The salivary gland microbiome profile consisted of *Pseudomonas* (29.32%), *Acinetobacter* (12.33%), *Staphylococcus* (12.1%), *Arcobacter* (9.55%), and *Serratia* (5.21%). The mid-gut microbiome profile consisted of *Pseudomonas* (29.11%), *Erwinia* (14.81%), *Staphylococcus* (8.89%), *Serratia* (8.61%), and *Pantoea* (6.03%) (Supplementary Information 3). Microbiome profiles of the mid-gut and salivary glands were evaluated based on their presence or absence in the samples (Fig. 4).

In both organs, the 21 genera commonly found, were found to be prevalent, with the mid-gut microbiome having more organ-specific taxa (20 genera) than the salivary glands (10 genera, Fig. 4A). Although organ-specific microbiotas such as *Arcobacter*, *Brevibacterium*, *Chryseobacterium*, *Asaia*, and *Enterobacteriaceae* were identified, only *Acinetobacter* has a statistically significant difference (Fig. 4B,C). Then, the differences by organ were compared by dividing the hyperendemic and hypoendemic areas. The dominant microbiome in the mid-gut and salivary gland were the same in the hyperendemic 6 area (*Serratia*) and all hypoendemic areas (*Pseudomonas*) (Table 2). However, the microbiome that dominated the mid-gut and salivary glands was different in hyperendemic areas (2–7) (Table 2). For example, *Staphylococcus* and *Brevibacterium* in hyperendemic area 1, *Pseudomonas*, and *Acinetobacter* in hyperendemic area 2 were dominant in the mid-gut and salivary glands, respectively (Table 2).

**Table 2:** Dominant microbiome by organ.

Area/organ	Mid-gut	Salivary glands
Hyperendemic area 1	*Staphylococcus* (88.32%)	*Brevibacterium* (43.81%)
Hyperendemic area 2	*Pseudomonas* (57.82%)	*Acinetobacter* (52.24%)
Hyperendemic area 3	*Erwinia* (56.76%)	*Staphylococcus* (27.57%)
Hyperendemic area 4	*Erwinia* (80.9%)	*Acinetobacter* (45.18%)
Hyperendemic area 5	*Enterobacteriaceae_uc* (62.15%)	*Acinetobacter* (38.46%)
Hyperendemic area 6	*Serratia* (82.68%)	*Serratia* (62.42%)
Hyperendemic area 7	*Pantoea* (53.51%)	*Staphylococcus* (68.51%)
Hyperendemic area 8	*AF166259_g* (31.1%)	*Arcobacter* (95.7%)
Hypoendemic areas	*Pseudomonas* (64.24%)	*Pseudomonas* (69.24%)

### Epidemiological differences in mosquito microbiome composition

The data was further compared based on patient incidence, which allowed for a division between hyperendemic and hypoendemic areas. There was a significant difference in alpha and beta diversity analyses based on epidemiology (Table 3, Figs.2, 6). The hyperendemic and hypoendemic areas were significant in PERMANOVA analysis using the Jaccard distance, which evaluates dissimilarity across data sets (Table 3). Furthermore, in both areas, the Shannon diversity index, which is a measure of the number (abundance) and relative abundance (uniformity) of species in the ecosystem, was significant (Fig. 2).

**Table 3:** *An.* *sinensis* microbiome pairwise beta diversity comparisons from different types of collecting sites.

Group 1	Group 2	Sample size	Permutations	pseudo-F	p-value	q-value
Hyperendemic area	Hypoendemic area	24	999	1.0521572475210700	0.017	0.017

The comparison of Jaccard distance in hyperendemic and hypoendemic areas revealed significant differences. However, The comparison of Bray–Curtis distance in hyperendemic and hypoendemic areas has no significant differences. PERMANOVA (999 permutations) tests with Benjamini–Hochberg FDR correction were used to make these comparisons (q-value). The significance level was set at $\hbox {q}<0.05$, with n = the number of pools processed and each pool containing 5 individual mosquitoes.

**Figure 5:**
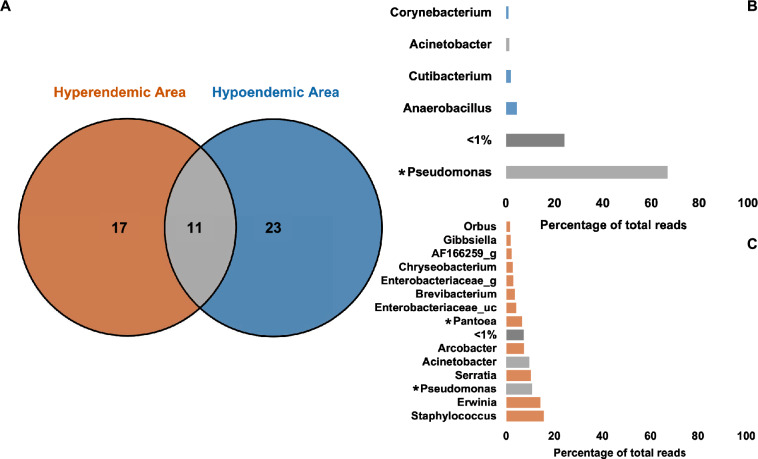
(**A**) Venn diagram analysis of shared genera by epidemiology. (**B**) Microbiome average profile of hyperendemic and (**C**) hypoendemic areas. Mosquitoes in hypoendemic and hyperendemic areas share 11 microbiotas, with 23 and 17 unique microbiotas identified in each. *Pseudomonas* is observed in both areas, which is much more prevalent in the hypoendemic area. *Staphylococcus* had the highest prevalence in the hyperendemic area, followed by *Erwinia*, *Pseudomonas*, *Serratia*, *Acinetobacter*, *Arcobacter*, and *Pantoea*. *Pseudomonas* and *Pantoea* were the statistically significant microbiotas.

Both areas shared 11 microbiotas mainly *Pseudomonas* and *Acinetobacter*. According to the analysis conducted, mosquitoes from hyperendemic areas exhibited 17 unique taxa, which were dominated by *Staphylococcus* (15.56%), *Erwinia* (14.05%), *Pseudomonas* (10.46%), *Serratia* (10.25%), *Acinetobacter* (9.48%) (Fig. 5). In hypoendemic areas, *Pseudomonas* (66.73%) dominates the microbiome profile, followed by *Anaerobacillus* (4.49%) (Fig. 5B). The microbiome profile of hyperendemic and hypoendemic areas have significant differences on *Pseudomonas* and *Pantoea* (Fig. 5B,C). *Pantoea* was only observed in hyperendemic areas (Fig. 5B,C). *Pseudomonas* was found at a low proportion in the hyperendemic area but was dominating in the hypoendemic area (Fig. 5B,C).

**Figure 6:**
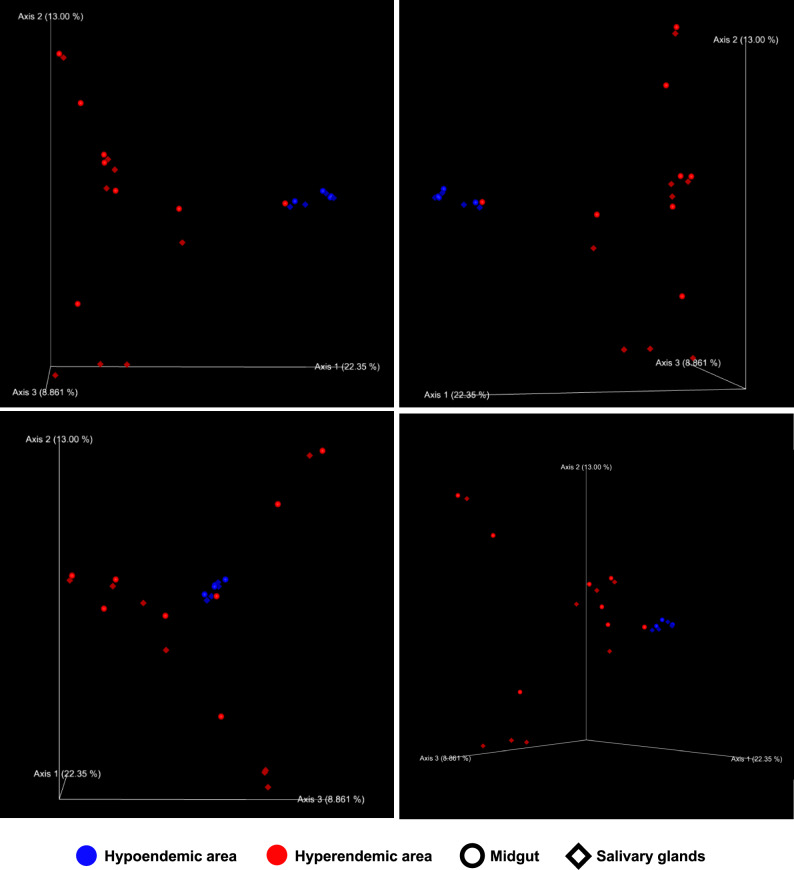
3D principal coordinated analysis (PCoA) plots of the Bray–Curtis distances of adult female *Anopheles sinensis* microbiome diversity according to epidemiology and organ This figure offers images that were shot in a multiple ranges of angles. The PCoA plot is a graph in which the distance between areas is estimated based on their dissimilarity in terms of relative abundance (Bray–Curtis). It can be seen that samples from the hyperendemic area are spread by a greater distance than those from the hypoendemic area, while samples from the hypoendemic area are closer together.

Principal coordinated analysis (PCoA) analysis showed great variance in microbial communities depending on epidemiology and collecting site (Fig. 6). In the graph, except for hyperendemic area 2-M, which is close to the sample of the hypoendemic area in graph, the hyperendemic area and hypoendemic area showed different patterns. Samples in the hypoendemic area were clustered regardless of region, but samples in the hyperendemic area had a clustering pattern according to region. In the hyperendemic area, 3 clustering were confirmed (Group 1: 1M, 1S, 3M, 3S, 7M, 7S, 8M, 8S, Group 2: 5M, 6M, 6S, Group 3: 2S, 4M, 4S, 5S) (Fig. 6). Group 1 shared *Erwinia*, *Pseudomonas*, and *Staphylococcus*, and Group 2 shared *Methylobacterium*, *Serratia*, and *Gibbsiella*. Group 3 shared *Pseudomonas*, *Acinetobacter*, *Pantoea*, and *Chryseobacterium* (Supplementary Information 3).

## Discussion

The definition of microbiome per se has recently expanded from microorganisms interacting with a host to the total of microbiota and their gene products related to environment and hosts^7^ influencing biology, longevity, behaviors, nutrition, and immunity^25–27^. The host’s microbiota has the potential to co-evolve and continually affect the host’s health^8^. There are several different environments where the microbiota may be acquired, including through food intake and wound invasion. As a result, the habitat of the host can affect the microbiome of the mosquito and may change based on regional environmental factors^27^. This study aimed to investigate whether there are significant differences in the microbiome profiles of mosquitoes between hyperendemic and hypoendemic areas of malaria incidence. The main goal of this study was to identify microbe species-biomarkers in different endemic areas which involved in malaria incidence. The current study demonstrates that the microbiome profile of mosquitoes collected in malaria-hyperendemic and hypoendemic areas was divided into distinct patterns at the level of genus and species. Mosquitoes in hyperendemic areas contained area-specific microbiome such as *Serratia*, *Staphylococcus*, *Erwinia*, *Enterobacter*, and *Arcobacter*, while *Pseudomonas* is dominant in hypoendemic areas, implying that malaria incidence can be analyzed with area-specific biomarkers. Remarkably, our findings revealed that the heterogeneity of microbiome profiles was higher in the hyperendemic area compared to the hypoendemic area, despite the spatial distance between hyperendemic areas being closer than in the hypoendemic area. It is suggested that malaria incidence in ROK might be related to northern border areas of malaria hot zones such as North Korea where malaria cases were reported in over 5000 patients in 2016^28^. As a result of human migration, cross-border population transfers, ecological changes, and vector population dynamics, border malaria frequently occurs in some transmission zones^29^. Malaria outbreaks on the Thai-Myanmar and Thai-Cambodia borders are typical border outbreaks, and they are caused by constant population migration, the movement of malaria patients, abuse of self-medication, and limitations on *Anopheles* vector management and surveillance^29^. In Africa, the patterns of malaria incidence are widespread especially in sub-Saharan desert areas by human migration and geographical changes such as dam construction, while malaria transmission in ROK is confined specifically to northern border areas^30^. This suggests that infected mosquitoes migrating from North Korea may be one of the major factors of malaria incidence in ROK, considering the restriction of human migration in the northern border areas of ROK. Therefore, it is important to trace the originality of malaria mosquitoes and their microbiome in hyperendemic areas by employing studies such as aerial netting methods to collect migrating mosquitoes at high altitudes from the north of DMZ, which could help to eradicate malaria in ROK^31^.

The microbiome of the hyperendemic and hypoendemic regions has high dissimilarity (Fig. 6). Nevertheless, this study faces certain limitations in comprehensively understanding the regional variation of mosquito microbiome within the hyperendemic area. Even though the hyperendemic 2-M sample was collected from a hyperendemic area, it had a microbiome that was similar to that found in samples collected from a hypoendemic area. Moreover, samples from the hyperendemic area showed three clustering, although this did not seem to be strongly related to the distance between each region. Further details on the environment of the region where the mosquito was collected (such as the mosquito’s aquatic habitat, blood sources, etc.) as well as whether the mosquito is *Plasmodium*-infected will be needed to comprehend the variation by region.

Interestingly, our study demonstrates that *Pseudomonas* is significantly different between the mosquito microbiome profiles of hyperendemic and hypoendemic areas, showing *Pseudomonas* was dominant in hypoendemic areas. *Pseudomonas* is commonly found in the gut microbiota of *Anopheles* mosquitoes^32–35^, indicating that *Pseudomonas* is capable of efficiently adapting to the mid-gut environment of *Anopheles* mosquitoes. In addition, a previous study has pinpointed that *Pseudomonas* is the most prevalent microbiota in the *Plasmodium*-negative groups^36^ and protects mosquitoes from the invasion of malaria parasites^37^. Interestingly, our study shows a low proportion of the *Psuedomonas* population of microbiome profiles in the hyperendemic areas, indicating that there might be some factors such as malaria parasites to disturb the balance of microbiota. It has been reported that *Pseudomonas* *synxantha* inhibits the growth of *Mycobacterium tuberculosis*^38^ causing tuberculosis, while little is known about the role of *Pseudomonas synxantha* in the mosquito. Having said that, it will be intriguing to elucidate the function of *Pseudomonas* which is the most dominant microbiome in hypoendemic areas in our study. Another focus of our study was the identification of species biomarkers specific to different endemic areas of malaria incidence in ROK. Therefore, further studies on spatial-temporal patterns of the mosquito microbiome are likely to be important to determine a seasonal variation of microbiome aligned with malaria incidence as well as tracing mosquito migration from elsewhere.

Although the major function of the mid-gut and salivary glands of mosquitoes is to digest diverse nutrients, these organs are crucial for the growth of gut-associated microbiota^39,40^ and pathogen transmission of mosquito-borne diseases^41–44^. The transmission of malaria parasites is dependent on maintaining parasite growth in the mid-gut and culminating virulent sporozoite stages in the salivary glands^45^. Therefore, the region-specific microbiome such as *Staphylococcus, Erwinia, Enterobacteriaceae, Serratia, Pantoea*, and *Acinetobacter* identified in each part of the hyperendemic area of this study has a potential role to interact with malaria parasites. *Serratia marcescens* is known to block the sporogonic development of *P. vivax* parasites in *An. albimanus*^46^, and *Acinetobacter* species increase the resistance of *An. gambiae* to *Plasmodium* development partly by the induction of anti-*Plasmodium* factors in Imd pathway^47^. Therefore, the potential roles of these region-specific microbiotas in the hyperendemic areas on the effect of malaria transmission remain to be investigated. In addition to the effects of the microbiota on parasite development, the microbiota is known to modulate host behaviors and thus increase vector competence. It has been reported that microbiome-Gut-Brain-Axis communication can change the sensory perception ability and blood-sucking behaviors of mosquitoes by specific neuropeptides secreted from certain microbiota^48^. Mosquitoes infected with malaria parasites show stronger blood-sucking behaviors, increasing the number of biting and precise finding of human hosts^49^, indicating that there might be some changes in microbiome-Gut-Brain-Axis communication in modified microbiota community. Interestingly, it has been demonstrated that the rapid proliferation of *Pseudomonas* in the mosquito mid-gut after blood-feeding induces the secretion of serotonin to suppress appetite, followed by the secretion of neuropeptide Y (NPY) in the mosquito brain^48,50,51^, which has pivotal roles in modulating sensory sensitivity such as olfaction^51^. Previous data indicate that one of the neuropeptides, tachykinin, also modulates olfactory pathways and insulin pathways in insects^52^ and synaptic contacts between terminals of tachykinergic neurons and NPY neurons regulate NPY neuronal activity in rats^53^, suggesting that neuropeptides secreted from microbiome can alter host finding behaviors of the mosquito by these neuronal pathways. Taken together, our study provides evidence that the microbiome composition in the mid-gut and salivary glands show spatially dynamic patterns, and it may influence the malaria incidence in ROK. Additional spatio-temporal variation of the microbiome will be providing more precise clues to understanding the origin of malaria mosquitoes in hyperendemic areas. It will be also interesting to elucidate the molecular and neuronal mechanisms of microbiome-Gut-Brain-Axis communication among host-microbiome-parasite underlying the modulation of host-finding behaviors. Although this study provides a snapshot of microbiome patterns in malaria-endemic areas in ROK, future studies will be required to estimate the *Plasmodium* concentration inside the mosquitoes samples to fully understand the exact patterns and dynamics of the microbiome in an individual mosquito, which could explain the correlation between *Plasmodium* spores and microbiome composition. Later on, fully understanding of microbiome patterns of malaria mosquitoes in the ROK and Korean peninsula will mitigate the efforts of the malaria eradication program.

## Methods

### Sample collection and identification

Female adult *Anopheles* *sinensis* mosquitoes were collected in 2020 (June 22 to 26) from 12 rural regions in ROK with the assistance of the regional center for vector surveillance against climate change supported by the Korea Centers for Disease Control and Prevention^54^. The collection sites were categorized as hyperendemic area if the number of malaria cases per 100,000 persons exceeds 1 patient. Otherwise, it was categorized as hypoendemic area (Fig. 1). The study regions were determined using statistics of malaria patient. The analysis could have improperly accounted for habitat or ecology since each region was not ecologically analyzed. Mosquitoes were sorted into genus using the morphological keys^55^, and species identifications were confirmed using a diagnostic PCR assay based on DNA barcode analysis^56^. Mosquito body parts such as legs and wings were transferred to a 1.5ml tube (Axygen, USA), after which DNA extraction was carried from mosquito parts using G-spin™ Total DNA Extraction kit (iNtRON’s, Korea) according to the manufacturer’s instructions. PCR cycle parameters involved an initial denaturation at 95 °C for 3 min, 35 cycles of 30 s at 95 °C, 30 s at 63 °C, and 2 min at 72 °C. A final extension at 72 °C for 10 min was completed. PCR products were subjected to electrophoresis on a 1.5% agarose gel and visualized under ultraviolet light.

### DNA extraction

The DNA extraction targeted Adult female mosquito mid-gut and salivary gland. Before to dissection, mosquitoes were surface sterilized for 1 min in 70% ethanol, then dissected in PBS (Phosphate-Buffered Saline). The dissecting stereo microscope working area was likewise sanitized with 70% ethanol during the dissection. mid-guts and salivary glands were pooled (each sample comprised of 5 mosquitoes) and kept at − 80 °C. Whole genome DNA was extracted under aseptic conditions using the Power soil kit (Qiagen, Germany) according to the manufacturer’s recommendations. Samples were used for 16S metagenomics analysis after DNA quality and quantity were checked. Two 16S rRNA-tagged libraries based on amplicons were created.

### 16S sequencing and taxonomic analysis

For all samples, National Instrumentation Center for Environmental Management (NICEM, Korea), performed PCR amplification, sample processing, and 16S rRNA gene sequencing (www.nicem.snu.ac.kr). The samples were amplified using the 2 × KAPA HiFi HotStart ReadyMix (Roche, Switzerland) and primers for the V3–V4 region of the 16S rRNA gene (Supplementary Information 5). The following were the PCR conditions: 3 min at 96 °C, then 30 cycles of 30 s at 96 °C, 30 s at 55 °C, 30 s at 72 °C, and finally 5 min at 72 °C. All the PCR results were then performed on 1.2% agarose gels to determine band size and intensity. Ampure XP beads (Beckman, USA) were used to purify amplified DNA from each sample. According to the content of DNA and molecular weight, samples were pooled in identical quantities and utilized to create Illumina DNA libraries. The libraries were then sequenced in Illumina MiSeq runs to obtain 2 × 300 bp paired-end reads. The paired-end sequencing reads were first imported into the QIIME2 v.2021.11^57^ pipelines for ‘quantitative insights into microbial ecology’, and the pipeline’s v.2021.11 was used to process and analyze the sequencing reads. The denoise-paired command was used to rectify errors, eliminate chimeras, and integrate paired-end reads using the DADA2 plugin in QIIME 2^58^. The sequence generated was utilized to compare the bacterial composition and taxonomy analysis later on. Moreover, chunlab analytical pipeline PKSSU 4.0 DB was used to group sequences at a 3% divergence (or 97% similarity) level, sequences were denoised and allocated to an operational taxonomic unit (OTU). Chimeric sequences that could not be definitively linked to an OTU were eliminated. OTUs that could not be taxonomically categorized were labeled “unclassified” and were excluded from further analysis. Phylum, genus, and species level data-sets were utilized for our analysis. At each level, count and ratio data are provided equally, and ratio data was used for analysis. For comparison, the ratio data were subdivided into epidemiology and organ (Supplementary Informations 2–4).

### Bacterial profiles

At the phylum, genus, and species levels, read count and abundance data for bacterial OTUs were evaluated. Low abundance taxa with a value of less than 1% were grouped into an “Other bacteria” category. EzBioCloud 16S database is designed to be best performed for species-level identification even though there is a limitation due to the lack of sequence differences in some closely related species. The combination of EzBioCloud 16S DB and sensitive bioinformatics pipelines allows us a species-level exploration of microbiome data.

### Species biomarker

Species biomarkers were used to find microbiota specific to each collection site. All analyses were conducted using “16S-based Microbiome Taxonomic Profiles (MTP)-Comparative Analyzer for MTP sets-Taxonomic biomarker discovery”, using the EzBioCloud application. A group containing samples from all regions and a group containing only samples from a specific region were compared using the Kruskal–Wallis H test, and among the microbiota with statistically significant values, the two or three microbiotas with the highest ratio were selected as species biomarkers (Supplementary Information 6).

### Statistical analysis

The Shannon diversity, Jaccard, and the Bray–Curtis dissimilarity index were used to analyze microbial diversity within (alpha diversity) and between (beta diversity) samples in QIIME2. The average Shannon indices that resulted were reported and compared between samples using pairwise Kruskal–Wallis tests with Benjamini–Hochberg FDR corrections for multiple comparisons. The results of the Jaccard and Bray–Curtis dissimilarity indices were compared between samples using paired PERMANOVA tests (999 permutations) with FDR corrections. The statistical analyses described above were performed using EZbiocloud^59^ (https://www.ezbiocloud.net/) and QIIME2^60^.

## Supplementary Information

Supplementary Information 1.

Supplementary Information 2.

Supplementary Information 3.

Supplementary Information 4.

Supplementary Information 5.

Supplementary Information 6.

## Data Availability

The metagenome reads obtained from this study have been deposited in NCBI under the BioProject PRJNA844511. All relevant data are either within the paper or will be submitted and will be available in a public repository at NCBI.

## References

[1] World Health Organization. *et al.* World malaria report 2020: 20 years of global progress and challenges. *World Malaria Report* (2020).

[2] Nadeem AY, Shehzad A, Islam SU, Al-Suhaimi EA, Lee YS (2022). Mosquirix™ rts, s/as01 vaccine development, immunogenicity, and efficacy. Vaccines.

[3] Ranson H, Lissenden N (2016). Insecticide resistance in African anopheles mosquitoes: A worsening situation that needs urgent action to maintain malaria control. Trends Parasitol..

[4] Yadav KK (2015). Molecular characterization of midgut microbiota of *Aedes albopictus* and *Aedes aegypti* from Arunachal Pradesh, India. Parasit. Vectors.

[5] Hegde S, Rasgon JL, Hughes GL (2015). The microbiome modulates arbovirus transmission in mosquitoes. Curr. Opin. Virol..

[6] Dennison NJ, Jupatanakul N, Dimopoulos G (2014). The mosquito microbiota influences vector competence for human pathogens. Curr. Opin. Insect Sci..

[7] Berg G (2020). Microbiome definition re-visited: Old concepts and new challenges. Microbiome.

[8] Guégan M (2018). The mosquito holobiont: Fresh insight into mosquito-microbiota interactions. Microbiome.

[9] Gao H, Cui C, Wang L, Jacobs-Lorena M, Wang S (2020). Mosquito microbiota and implications for disease control. Trends Parasitol..

[10] Coon KL, Brown MR, Strand MR (2016). Gut bacteria differentially affect egg production in the anautogenous mosquito *Aedes aegypti* and facultatively autogenous mosquito *Aedes atropalpus* (diptera: Culicidae). Parasit. Vectors.

[11] Sharma A, Dhayal D, Singh O, Adak T, Bhatnagar RK (2013). Gut microbes influence fitness and malaria transmission potential of Asian malaria vector *Anopheles stephensi*. Acta Trop..

[12] Dutra HLC (2016). Wolbachia blocks currently circulating zika virus isolates in Brazilian *Aedes aegypti* mosquitoes. Cell Host Microb..

[13] Moreira LA (2009). A wolbachia symbiont in *Aedes aegypti* limits infection with dengue, chikungunya, and plasmodium. Cell.

[14] Utarini A (2021). Efficacy of wolbachia-infected mosquito deployments for the control of dengue. N. Engl. J. Med..

[15] Ramirez JL (2014). Chromobacterium csp\_p reduces malaria and dengue infection in vector mosquitoes and has entomopathogenic and in vitro anti-pathogen activities. PLoS Pathog..

[16] Ryan PA (2019). Establishment of wmel wolbachia in *Aedes aegypti* mosquitoes and reduction of local dengue transmission in cairns and surrounding locations in northern Queensland, Australia. Gates Open Res..

[17] Boissière A (2012). Midgut microbiota of the malaria mosquito vector *Anopheles gambiae* and interactions with *Plasmodium falciparum* infection. PLoS Pathog..

[18] Osei-Poku J, Mbogo C, Palmer W, Jiggins F (2012). Deep sequencing reveals extensive variation in the gut microbiota of wild mosquitoes from Kenya. Mol. Ecol..

[19] Zouache K (2011). Bacterial diversity of field-caught mosquitoes, *Aedes albopictus* and *Aedes aegypti*, from different geographic regions of Madagascar. FEMS Microbiol. Ecol..

[20] Valiente Moro C, Tran FH, Nantenaina Raharimalala F, Ravelonandro P, Mavingui P (2013). Diversity of culturable bacteria including pantoea in wild mosquito *Aedes albopictus*. BMC Microbiol..

[21] Ngo CT, Romano-Bertrand S, Manguin S, Jumas-Bilak E (2016). Diversity of the bacterial microbiota of anopheles mosquitoes from Binh Phuoc province, Vietnam. Front. Microbiol..

[22] Organization WH (1981). Synopsis of the world malaria situation, 1979. Wkly. Epidemiol. Rec..

[23] Chai J-Y (1999). Re-emerging *Plasmodium vivax* malaria in the Republic of Korea. Korean J. Parasitol..

[24] Wangdi K, Gatton ML, Kelly GC, Clements AC (2015). Cross-border malaria: A major obstacle for malaria elimination. Adv. Parasitol..

[25] Azambuja P, Garcia ES, Ratcliffe NA (2005). Gut microbiota and parasite transmission by insect vectors. Trends Parasitol..

[26] Coon KL, Vogel KJ, Brown MR, Strand MR (2014). Mosquitoes rely on their gut microbiota for development. Mol. Ecol..

[27] Yun J-H (2014). Insect gut bacterial diversity determined by environmental habitat, diet, developmental stage, and phylogeny of host. Appl. Environ. Microbiol..

[28] Kim J-H, Lim A-Y, Cheong H-K (2019). Malaria incidence of the regions adjacent to the demilitarized zone in the democratic people’s Republic of Korea, 2004–2016. J. Korean Med. Sci..

[29] Bhumiratana A, Intarapuk A, Sorosjinda-Nunthawarasilp P, Maneekan P, Koyadun S (2013). Border malaria associated with multidrug resistance on Thailand-Myanmar and Thailand-Cambodia borders: Transmission dynamic, vulnerability, and surveillance. BioMed Res. Int..

[30] Kibret S, Lautze J, McCartney M, Wilson GG, Nhamo L (2015). Malaria impact of large dams in Sub-Saharan Sfrica: Maps, estimates and predictions. Malar. J..

[31] Chapman J, Reynolds D, Smith A, Smith E, Woiwod I (2004). An aerial netting study of insects migrating at high altitude over England. Bull. Entomol. Res..

[32] Rani A, Sharma A, Rajagopal R, Adak T, Bhatnagar RK (2009). Bacterial diversity analysis of larvae and adult midgut microflora using culture-dependent and culture-independent methods in lab-reared and field-collected anopheles stephensi-an Asian malarial vector. BMC Microbiol..

[33] Chavshin AR (2014). Isolation and identification of culturable bacteria from wild *Anopheles culicifacies*, a first step in a paratransgenesis approach. Parasit. Vectors.

[34] Pumpuni CB, Demaio J, Kent M, Davis JR, Beier JC (1996). Bacterial population dynamics in three anopheline species: The impact on *Plasmodium sporogonic* development. Am. J. Trop. Med. Hyg..

[35] Chavshin AR (2012). Identification of bacterial microflora in the midgut of the larvae and adult of wild caught *Anopheles stephensi*: A step toward finding suitable paratransgenesis candidates. Acta Trop..

[36] Oliveira TMP, Sanabani SS, Sallum MAM (2020). Bacterial diversity associated with the abdomens of naturally plasmodium-infected and non-infected *Nyssorhynchus darlingi*. BMC Microbiol..

[37] Feng Y (2022). Anopheline mosquitoes are protected against parasite infection by tryptophan catabolism in gut microbiota. Nat. Microbiol..

[38] Mukherjee K, Mandal S, Mukhopadhyay B, Mandal NC, Sil AK (2014). Bioactive compound from *Pseudomonas synxantha* inhibits the growth of mycobacteria. Microbiol. Res..

[39] Dillon RJ, Dillon V (2004). The gut bacteria of insects: Nonpathogenic interactions. Annu. Rev. Entomol..

[40] Oliveira JHM (2011). Blood meal-derived heme decreases ros levels in the midgut of *Aedes aegypti* and allows proliferation of intestinal microbiota. PLoS Pathog..

[41] James A, Rossignol P (1991). Mosquito salivary glands: Parasitological and molecular aspects. Parasitol. Today.

[42] Sim S, Ramirez JL, Dimopoulos G (2012). Dengue virus infection of the *Aedes aegypti* salivary gland and chemosensory apparatus induces genes that modulate infection and blood-feeding behavior. PLoS Pathog..

[43] Fontaine A (2011). Implication of haematophagous arthropod salivary proteins in host-vector interactions. Parasit. Vectors.

[44] Browne, L. B. Regulatory mechanisms in insect feeding. In *Advances in Insect Physiology*, vol. 11, 1–116 (Elsevier, 1975).

[45] Snow RW (1998). Models to predict the intensity of *Plasmodium falciparum* transmission: Applications to the burden of disease in Kenya. Trans. R. Soc. Trop. Med. Hyg..

[46] Gonzalez-Ceron L, Santillan F, Rodriguez MH, Mendez D, Hernandez-Avila JE (2003). Bacteria in midguts of field-collected *Anopheles albimanus* block plasmodium vivax sporogonic development. J. Med. Entomol..

[47] Bahia AC (2014). Exploring a nopheles gut bacteria for plasmodium blocking activity. Environ. Microbiol..

[48] De Das T (2022). Bidirectional microbiome-gut-brain-axis communication influences metabolic switch-associated responses in the mosquito *Anopheles culicifacies*. Cells.

[49] Stanczyk NM, Mescher MC, De Moraes CM (2017). Effects of malaria infection on mosquito olfaction and behavior: Extrapolating data to the field. Curr. Opin. Insect Sci..

[50] Jenkins TA, Nguyen JC, Polglaze KE, Bertrand PP (2016). Influence of tryptophan and serotonin on mood and cognition with a possible role of the gut-brain axis. Nutrients.

[51] Duvall LB, Ramos-Espiritu L, Barsoum KE, Glickman JF, Vosshall LB (2019). Small-molecule agonists of *Ae. aegypti* neuropeptide y receptor block mosquito biting. Cell.

[52] Jung JW (2013). Neuromodulation of olfactory sensitivity in the peripheral olfactory organs of the American cockroach, *Periplaneta americana*. PLoS ONE.

[53] Magoul R, Tramu G (2000). Tachykinin-induced stimulation of neuropeptide y gene expression in the rat arcuate nucleus. Neuroreport.

[54] Lee W, Yang S (2014). Introduction of regional center for vector surveillance against climate change. Korea KDCA Public Health Wkly. Rep..

[55] Ree H-I (2003). Taxonomic review and revised keys of the Korean mosquitoes (diptera: Culicidae). Entomol. Res..

[56] Joshi D, Park M, Saeung A, Choochote W, Min G (2010). Multiplex assay to identify Korean vectors of malaria. Mol. Ecol. Resourc..

[57] Bolyen E (2019). Reproducible, interactive, scalable and extensible microbiome data science using qiime 2. Nat. Biotechnol..

[58] Callahan BJ (2016). Dada2: High-resolution sample inference from illumina amplicon data. Nat. Methods.

[59] Yoon S-H (2017). Introducing ezbiocloud: A taxonomically united database of 16S rrna gene sequences and whole-genome assemblies. Int. J. Syst. Evol. Microbiol..

[60] Hall, M. & Beiko, R. G. 16S rrna gene analysis with qiime2. In *Microbiome analysis*, 113–129 (Springer, 2018). 10.1007/978-1-4939-8728-3_8 30298251

